# De-escalation of adjuvant radio(chemo)therapy for patients with HPV-positive head and neck squamous cell carcinoma: study protocol for a phase I trial to reduce late toxicity (DELPHI)

**DOI:** 10.1186/s12885-026-16050-x

**Published:** 2026-04-29

**Authors:** Annett Linge, Fabian Lohaus, Steffen Appold, Esther G.C. Troost, Amir Abdollahi, Susann Kowal, Daniel Büttner, Steffen Löck, Michael Baumann, Mechthild Krause

**Affiliations:** 1 Department of Radiotherapy and Radiation Oncology, Faculty of Medicine and University Hospital Carl Gustav Carus, TUD Dresden University of Technology, Dresden, Germany; 2 OncoRay – National Center for Radiation Research in Oncology, Faculty of Medicine and University Hospital Carl Gustav Carus TUD Dresden University of Technology, Dresden, Germany; 3 German Cancer Research Center (DKFZ), Heidelberg, Germany, and German Cancer Consortium (DKTK), Partner site Dresden, Dresden, Germany; 4 National Center for Tumor Diseases (NCT), NCT/UCC Dresden, a partnership between DKFZ, Faculty of Medicine and University Hospital Carl Gustav Carus, TUD Dresden University of Technology, and Helmholtz-Zentrum Dresden-Rossendorf (HZDR), Dresden, Germany; 5 Helmholtz-Zentrum Dresden-Rossendorf, Institute of Radiooncology – OncoRay, Dresden, Germany; 6 Clinical Cooperation Unit Translational Radiation Oncology, German Cancer Consortium (DKTK) Core-Center Heidelberg, National Center for Tumor Diseases (NCT), Heidelberg University Hospital (UKHD) and German Cancer Research Center (DKFZ), Heidelberg, Germany; 7 Division of Molecular and Translational Radiation Oncology, Heidelberg Faculty of Medicine (MFHD) and Heidelberg University Hospital (UKHD), Heidelberg Ion-Beam Therapy Center (HIT), Heidelberg, Germany; 8 Heidelberg Institute of Radiation Oncology (HIRO), National Center for Radiation Oncology (NCRO), Heidelberg University Hospital and German Cancer Research Center (DKFZ), Heidelberg, Germany; 9 National Center for Radiation Research in Oncology (NCRO), Heidelberg Institute for Radiation Oncology (HIRO), Heidelberg, Germany; 10 German Cancer Research Center (DKFZ), and German Cancer Consortium (DKTK), Core Center Heidelberg, Heidelberg, Germany; 11 German Cancer Research Center (DKFZ) Heidelberg, Division of Radiooncology / Radiobiology, Heidelberg, Germany

**Keywords:** Dose de-escalation, HNSCC, HPV, Oropharyngeal carcinoma, Phase I clinical trial, Radiotherapy

## Abstract

**Background:**

Patients with locally advanced head and neck squamous cell carcinoma are receiving adjuvant radio(chemo)therapy as standard of care, according to national guidelines. However, patients with human papilloma virus (HPV) driven oropharyngeal squamous cell carcinoma (OPSCC), are shown to have superior locoregional control (LRC) rates, suggesting that they are likely being overtreated. To date it is unknown, if and to which extent adjuvant radiotherapy can be safely reduced.

**Methods and design:**

The interventional multicentric DELPHI trial is investigating step-wise radiation dose reduction in patients with both p16-overexpressing and HPV16 DNA positive OPSCC. Depending on international clinical and histopathological risk factors, patients are being enrolled in the high-risk or intermediate-risk arm. Patients of the high-risk arm are receiving standard simultaneous chemotherapy with cisplatin. Patients with smoking history of at least 30 packyears are being treated in the observational arm.

Primary endpoint of the DELPHI trial is LRC after 24 months. Secondary endpoints are acute and late toxicity, quality of life during and up to 24 months after the end of therapy as well as LRC and overall survival after 60 months.

**Discussion:**

Primary aim of the DELPHI trial is to show that radiation dose reduction is safe and therefore feasible in patients with HPV-positive OPSCC. Secondary objective is to show that radiation-dose reduction leads to less late toxicity compared with standard treatment and thus improves quality of life.

**Trial registration:**

The DELPHI trial is registered at clinicaltrials.gov under the identifier NCT03396718.

**Supplementary Information:**

The online version contains supplementary material available at 10.1186/s12885-026-16050-x.

## Introduction/ rationale

Patients with locally advanced, resectable head and neck squamous cell carcinoma are receiving surgery, followed by standard adjuvant radio(chemo)therapy according to national and international guidelines. This has been established stepwise. First, it has been shown that dose escalation to the previous tumour bed as well as to cervical levels with lymph node metastases results in higher local control rates in patients with microscopical residual disease [1, 2]. However, dose escalation to more than 57 Gy was only beneficial in patients who are on high risk to develop recurrences, i.e. those with residual disease, large tumours, more than two regional lymph node metastases or lymph nodes with extracapsular extension (ECE). To further improve local control, the effect of simultaneous chemotherapy was studied within two clinical trials of the EORTC and the RTOG, which led to higher locoregional control rates, extended disease-free survival and longer overall survival of patients who received combined radiochemotherapy in comparison to those with adjuvant radiotherapy alone [3, 4]. This led to the current standard therapy of patients who are on high risk to develop recurrences, with doses up to 66 Gy and concurrent cisplatin-based chemotherapy. However, with increasing total radiation dose to the swallowing apparat and to the salivary glands the incidence of high grade late effects is rising [5, 6].

Well-known risk factors for the induction of HNSCC are smoking and alcohol consumption. In addition, infection with human papillomavirus (HPV) has been shown to particularly induce oropharyngeal squamous cell carcinoma (OPSCC) and over the last two decades the incidence of HPV-positive tumours has been increasing in the United States and in Europe [7, 8]. Furthermore, it has been shown that patients with HPV-positive disease have superior oncological outcomes and are also characterized by higher response rates after primary radiochemotherapy [9–14] and after adjuvant radiochemotherapy [15]. In Germany, patients with HPV-positive OPSCC often receive primary resection and, patients with high risk for recurrences according to established clinical risk factors, adjuvant radio(chemo)therapy. However, this multimodal treatment approach may lead to extensive late toxicities. While patients with HPV-positive OPSCC have a favourable prognosis and very high response rates to radio(chemo)therapy [9–14], thus likely becoming long-term survivors, they suffer from their chronic late toxicities.

Within the last decade, de-escalation of simultaneous chemotherapy has been investigated within clinical trials, with the overall aim to decrease toxicity in patients with OPSCC while maintaining high overall survival rates. However, after two randomised controlled clinical phase III trials showed a significant decrease in overall survival [16, 17] when replacing concomitant cisplatin by cetuximab without significantly reducing toxicity, cisplatin currently remains the standard therapy in patients with high-risk OPSCC.

The reduction of radiation dose with the overall aim to decrease chronic toxicities while maintaining excellent tumour control seems to be a promising strategy for improving therapeutic ratio in patients who are receiving adjuvant radio(chemo)therapy. A step-wise dose reduction within clinical trials is a straight-forward approach to find the optimal radiation dose for adjuvant treatment, which can then be studied within a randomised clinical trial in comparison to the current standard radio(chemo)therapy. Within the last years, clinical trials demonstrated that radiation dose de-escalation can be performed while maintaining high local control rates in patients with HPV-positive OPSCC. In a phase III study by Ferris et al. [18] in patients with intermediate risk for recurrences, dose reduction from 60 Gy to 50 Gy led to similar local control rates with 96% vs. 94,9% after 2 years. The AVOID phase II study in OPSCC patients with completely resected tumours (negative surgical margins at least 2 mm, no evidence of lymphovascular or perineural invasion) but lymph node metastases studied volume and dose de-escalation with respect to the previous tumour region. They showed that omission of adjuvant radiotherapy of the resected primary tumour region, while keeping the dose to the uninvolved neck regions (54 Gy total dose) and irradiation of the involved neck regions (60 to 66 Gy) leads to LRC of 98,3% after 2 years [19]. In a phase II study by Ma et al. [20], risk-adapted dose de-escalation to 30 to 36 Gy showed local control rates of about 96% after 2 years, which is comparable to historical control rates. Their recently published phase III trial provided randomised evidence that this substantial dose reduction resulted in decreased late toxicity [21]. However, this trial needs to be interpretated with caution. It was powered to detect differences in side effects, not oncological outcomes, and thus the trial does not allow to conclude that dose de-escalation to 30–36 Gy is safe in terms of disease control. Other clinical studies on dose- and/or volume-based de-escalation of adjuvant radiotherapy in patients with OPSCC are currently ongoing, e.g. as reviewed in [22, 23].

Proton therapy may also be an option to achieve high response rates with lower doses to organs at risk and therefore a potential reduction in late toxicities. Even though proton therapy is currently not widely available, it is allowed within the DELPHI trial presented here in order to open the possibility for comparison between both treatment modalities.

As mentioned above, smoking is one of the risk factors for the development of HNSCC. According to Ang et al. [24], cumulative nicotine abuse of more than 10 years may be associated with lower local control rates. However, this could not be confirmed by epidemiological studies or clinical data sets [25–27]. Within the current study, patients with a cumulative nicotine abuse of at least 30 packyears are being excluded from dose de-escalation and are enrolled in the observational arm.

Overall, patients with HPV-positive OPSCC are characterised by higher local control rates in comparison to those with HPV-negative disease. The hypothesis of our study is that moderate dose reduction does not lead to an increase of locoregional recurrences but results in less late side effects. Within this study, the HPV status is analysed centrally and includes p16 immunohistochemistry and HPV DNA genotyping. HPV-positive tumours are characterised by p16 overexpression of at least 70% of the tumour cells and HPV16 DNA positivity to ensure as much as possible that patients with truly HPV-driven tumour are being enrolled in the interventional arms [28].

Nevertheless, the potential risk of increased recurrences cannot be fully ruled out and is being monitored by several instances, including dummy runs of a treatment plan, quality control and central review of the first three treatment plans per center, central HPV analysis, stopping criteria of the study and step-wise reduction of the total dose including a monitoring time of 24 months of patients with intermediate- as well as in patients with high-risk disease.

The study protocol has been prepared in accordance with the SPIRIT (Standardised Protocol Items: Recommendation for Interventional Trials) guidelines [29].

## Study design

This is a multicentric, non-randomised phase I study, conducted at multiple academic university hospitals in Germany with expertise in head and neck oncology and radiotherapy. It consists of two interventional treatment arms and step-wise dose-reduction depending on risk factors for the development of recurrences. Allocation to the study arms is based on predefined clinical and histopathological risk factors and HPV status, as assessed centrally. Patients are enrolled in the high-risk arm if at least one of the following criteria is met: microscopically residual disease after resection, ECE of one or more lymph node metastases or large tumours (pT4) and a minimum of three lymph node metastases. For the intermediate-risk arm, patients with pT3 tumours or one to three lymph node metastases without any of the risk factors of the high-risk profile are included. Due to the nature of the intervention, blinding of participants and treating physicians is not feasible. The inclusion and exclusion criteria are summarised in Table 1. The schedule of enrolment, interventions and assessments is shown in the Supplementary figure.

**Table 1:** Inclusion and exclusion criteria

Inclusion criteria:
• Oropharyngeal squamous cell carcinoma with indication of adjuvant radio(chemo)therapy
• ECOG performance status 0-1
• Experimental arm: HPV16(+) tumours
• Observational arm: HPV16(-) tumours
• Adequate compliance to ensure tumour follow-up care as per study protocol
• Age ≥ 18 years
• Written informed consent
• Neck dissection of at least tumour-bearing side
**Additional inclusion criteria, high risk arm (at least 1 factor to be fulfilled):**
• R1 resection and/or
• pT4 status and/or
• > 3 lymph node metastases and/or
• Extracapsular spread of lymph node metastasis
**Additional inclusion criteria for intermediate risk arm (at least 1 factor to be fulfilled):**
• pT3R0 and/or
• 1-3 lymph node metastases and no extracapsular spread of lymph node metastasis
Exclusion criteria:
• Cumulative nicotine abuse > 30 packyears for intervention arms. These patients are always included in control arms (regardless of HPV status).
• M1
• R2 resection
• No neck dissection performed
• >7 weeks interval surgery-radiotherapy
• Contraindication for guideline-compliant adjuvant radio(chemo)therapy according to clinical risk constellation
• Malignant tumour disease in the last five years before start of study (except basal cell carcinoma of the skin, in-situ carcinoma of the cervix uteri or breast or tumours with a similar good prognosis that are considered very likely to be cured)
• Any malignant tumour disease in the head and neck region regardless of interval and prognosis
• Previous radiotherapy with potential overlap
• Participation in another clinical trial if, and as result of this, another experimental therapy is necessary or the therapies/study protocols are mutually exclusive (e.g. modified chemotherapy, additional consolidating chemotherapy; immunotherapy). Additional participation in observational studies or supportive therapy studies are allowed
• Any other diseases or conditions that do not allow the patient to assess the nature and scope as well as possible consequences of the clinical study
• Pregnant or breastfeeding women

Dose de-escalation is performed in two steps with a dose-reduction by 10% each. The second dose de-escalation step is opened, when all of the planned 30 patients were accrued to the respective interventional arm, when at least 10 out of 30 patients finished their 24 months follow-up time and if the first stopping criterium (two recurrences within 24 months follow-up among the first 10 patients in the respective interventional arm) has not been reached. Patients enrolled after closure of the de-escalation arms, are being included in the observational arm and receive standard radio(chemo)therapy.

**Fig. 1:**
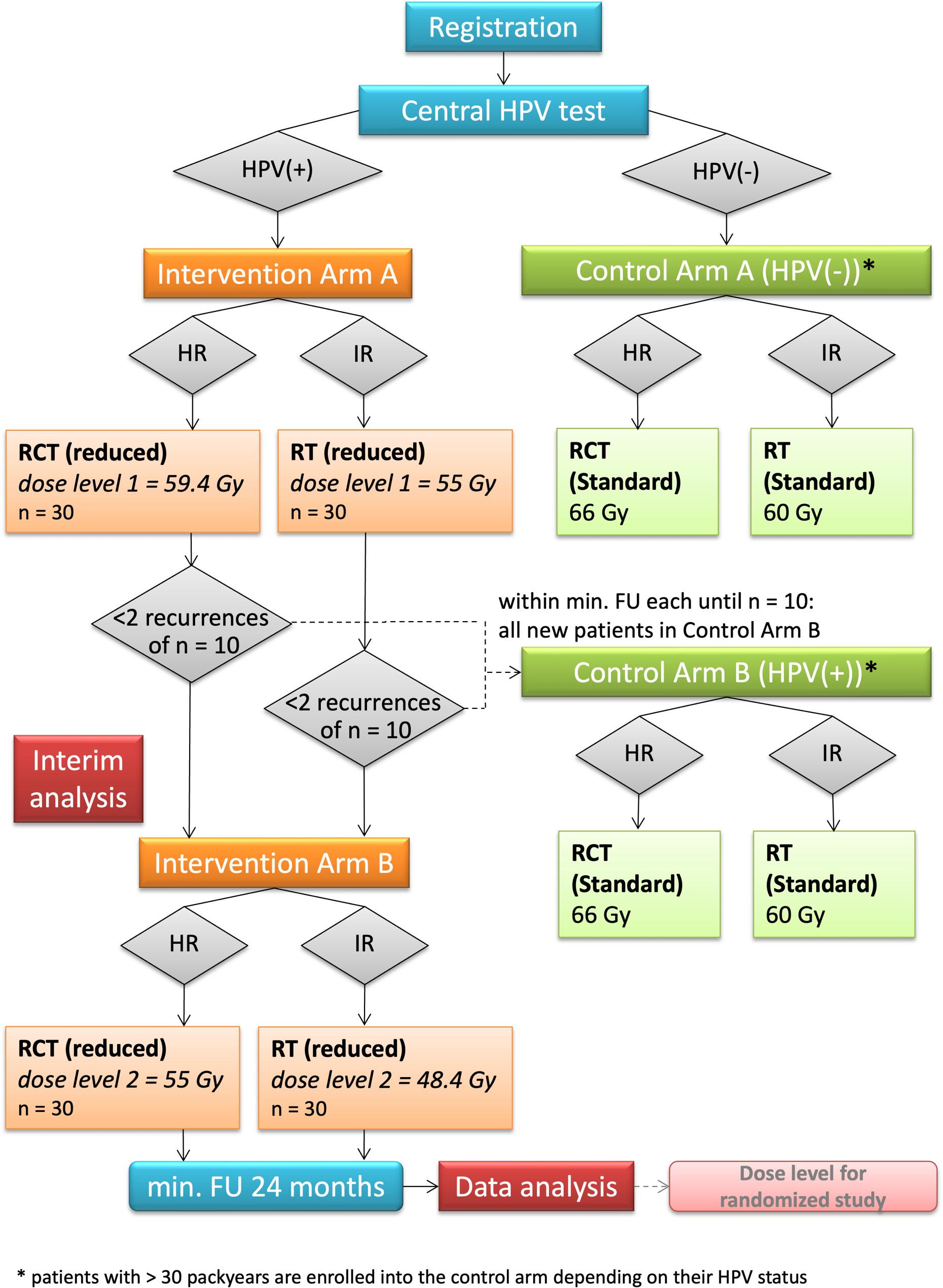
Flowchart of the DELPHI trial

The flowchart of the study is depicted in Fig. 1. Patients with operable OPSCC and indication for adjuvant radio(chemo)therapy, as recommended by the experts in the interdisciplinary tumour board for malignancies of the head and neck, are offered to participate in this clinical trial. After patient’s consent and consultation, FFPE tumour material is analysed centrally at the Institute of Pathology at the University Hospital Dresden and includes HPV analysis using p16 immunohistochemistry and PCR-array based HPV DNA genotyping. Patients with proven HPV-association of their tumours, e.g. positivity for both p16 (defined as continuous, strong block-type p16-positivity of the cytoplasm and/or nuclei in at least 70% of the tumour cells [24]) and HPV16 DNA (polymerase chain reaction (PCR)-array based analysis), are subjected to the interventional arm according to their risk for recurrences [intermediate-risk (IR) vs. high-risk (HR)] and treated with 10% dose reduction to both the former tumour bed and the lymph node levels. Patients without HPV-associated tumours are being allocated to the observational arm and are receiving standard radio(chemo)therapy within this prospective trial. After enrollment of 30 patients per risk-arm, patients are also being enrolled in the observational arm A or B (according to their risk profile) until a minimum follow-up of 2 years has been reached for 10 patients for the respective arm. For these patients, HPV is assessed but standard radio(chemo)therapy is applied irrespective of its result. The cohorts of the observational arms are serving as comparison group.

For subgrouping of the patients according their clinical and histological risk factors and the HPV status of their tumours, both clinical factors as well as HPV results are recorded in electronic case report forms (eCRFs) within the central RadPlanBio platform [30, 31], which is accessible from all participating study centers.

### Treatment description

Patients are being treated at the respective participating center of this trial. Every patient receives a planning CT scan in treatment position for 3-dimensional treatment planning with a clinically approved treatment planning system. All patients receive an individual thermoplastic radiotherapy mask for positioning and will be treated with image-guided, intensity-modulated radiotherapy (IMRT), or alternatively, with intensity-modulated proton therapy. The scar from the neck dissection should be marked by radiopaque material prior to the planning CT scan. IMRT can be performed as static (step&shoot) or rotational irradiation (RapidArc, Volumetric Modulated Arc Therapy).

Contouring of the lymph node regions is being performed according to actual guidelines of the international committees [32, 33].

Two target volumes are defined: planning target volume_adjuvant (PTV_adjuvant) and PTV_boost. Organs of risk to be included and contoured are the spinal cord, brain stem, parotid glands and submandibular glands.

Dose prescription is to a representative point in the target volume, according to ICRU report 78. In addition, the maximal dose in the target volume (Dmax) should not exceed 110% of the prescribed dose in the PTV_boost, and the minimal dose (Dmin) should not be below 95% of the prescribed dose but must be 90% of the prescribed dose at minimum. The volume within the PTV_adjuvant, that receives >2 Gy/fraction, i.e. >110% of the prescribed dose, should be as small as possible.

The target volume doses for all treatment arms and dose de-escalation steps are depicted in Table 2.

**Table 2:** Prescribed doses in the study arms

1st dose de-escalation step			
**Treatment arm**	**Number of fractions**	**Dose per fraction**	**Final dose**
High risk	25	1.8 Gy/ 2.2 Gy*	45.0 Gy (adjuvant volume) / 55.0 Gy (boost volume)
	2	2.2 Gy**	59.4 Gy (boost volume)
Intermediate risk	25	1.8 Gy / 2.2 Gy*	45.0 Gy (adjuvant volume) / 55.0 Gy (boost volume)
2nd dose de-escalation step			
**Treatment arm**	**Number of fractions**	**Dose per fraction**	**Final dose**
High risk	22	1.8 Gy/ 2.2 Gy*	39.6 Gy (adjuvant volume) / 48.4 Gy (boost volume)
	3	2.2 Gy**	55.0 Gy (boost volume)
Intermediate risk	22	1.8 Gy / 2.2 Gy*	39.6 Gy (adjuvant volume) /48.4 Gy (boost volume)
Observational study arm^#^			
**Treatment arm**		**Dose per fraction**	**Final dose**
High risk		1.8 - 2.0 Gy	50.0 - 50.4 Gy (adjuvant volume)
		1.8 - 2.2 Gy***	64.0 – 66.0 Gy (boost volume)
Intermediate risk		1.8 - 2.0 Gy	50.0 - 50.4 Gy (adjuvant volume)
		1.8 - 2.2 Gy***	58.0 – 60.0 Gy (boost volume)

*applied as simultaneous integrated boost (SIB); ** applied as sequential boost; ***applied as sequential or simultaneous boost; #In the observational study arm, dose prescription can also performed as per standard operating procedure of the respective centre

For the CTV_boost (irrespective of the treatment arm), the previous tumour region is contoured considering initial imaging performed prior to any treatment (computed tomography, magnetic resonance tomography, positron emission computed tomography), initial pan endoscopy, surgery operation reports and any histopathological reports. In case of lymph node metastases, the respective lymph node level is included in the treatment volume (CTV_boost). The CTV_boost as well as the CTV_adjuvant should be 5 mm below skin at maximum.

The CTV_adjuvant includes the CTV_boost and the elective lymph node regions II to IVa on both sides, and Level Va, Vb and VIIa at the tumour-bearing side and is being further defined as summarised in Table 3. The PTVs are applied using centre-specific margins.

**Table 3:** Definition of the adjuvant clinical target volume (CTV)

Definition of the adjuvant CTV:
• The adjuvant volume consists of the CTV_boost and the elective lymph node regions II-IVa (both sides) and level Va, Vb and VIIa of the tumour-bearing side.
**In addition (all study arms):**
• If Level II is affected, level IB will also be included.
• In case of tongue infiltration, level IB will also be included.
• If Level II is affected, the affected side will be included up to the base of the skull (level VIIa and VIIb).
• If the contralateral side is affected, level Va and Vb contralateral will be included.
• If level IVa is affected, level IVb and Vc will also be included.
**In addition for the intermediate-risk arm only:**
• If pN0, neck-dissection of both sides and strict lateral tumour localisation (no infiltration of the ground of the tongue, no infiltration of the uvula (for tumours of the soft palate)) of the tumour of the tonsil, one-sided irradiation of the lymph nodes is performed.
• In case of an ipsilateral lymph node metastasis in level II, neck-dissection of both sides and strict lateral tumour localisation (no infiltration of the ground of the tongue, no infiltration of the uvula (for tumours of the soft palate)) of the tumour of the tonsil, one-sided irradiation of the lymph node levels is performed.
• If Level II is affected, level IB will also be included.

Doses to organs at risk are based on the QUANTEC analysis [5, 34, 35]. The constraints for the maximal dose to the spinal cord (Dmax 45 Gy) and to the brain stem (Dmax 54 Gy) have to be met in all cases. Constraints for the parotid glands should be kept, but only when not compromising the prescribed dose to the CTV. The contralateral parotid gland should receive a median dose below 20 Gy, but in general a median dose as low as possible. 

Irradiation is being performed once daily, 5 times per week. Treatment breaks are not intended and not planned, i.e. treatment breaks have to be compensated after failure of more than 1 fraction at latest. Compensation irradiation can be applied at the weekend or as a second fraction on a day during the week. However, the interval between 2 fractions should be at least 6 h, but favourably 8 h. Not more than 6 fractions per week should be given.

Treatment modifications or discontinuation will be performed at the discretion of the treating physician in cases of unacceptable toxicity, patient request, or medical contraindications, and will be documented accordingly.

### Assessment of toxicities and quality of life

Therapy-related side effects are being scored according to the Common Terminology Criteria for Adverse Events v4.0 (CTCAE 4.0), early and late side effects of the skin are being scored according to the guidelines of the RTOG (Radiation Therapy Oncology Group, http://www.rtog.org/ResearchAssociates/AdverseEventReporting/AcuteRadiationMorbidityScoringCriteria.aspx and http://www.rtog.org/ResearchAssociates/AdverseEventReporting/RTOGEORTCLateRadiationMorbidityScoringSchema.aspx).

Toxicity is scored at study visits before start of radio(chemo)therapy, once per week during radio(chemo)therapy and at the end of radio(chemo)therapy. Thereafter, scoring is performed after 6 weeks, every 3 months within the first 2 years and then every 6 months up to 60 months after end of radio(chemo)therapy. Side effects are being documented as new side effects at their first occurrence, or if an increased grade of a previously recorded side effect has been documented. Furthermore, tumour follow-up care is being scheduled at each of the study visits starting 3 months after end of radio(chemo)therapy at the respective head and neck department. In addition, radiological imaging is being performed regularly after end of therapy.

Patients are being educated regarding the side effects using standardized educational documents for radiotherapy or radiochemotherapy. It is expected, that patients suffer from less side effects in the interventional treatment arms compared to the standard treatment.

All patients of the high-risk arms are receiving simultaneous chemotherapy with cisplatin as per standard protocol, aiming to reach a cumulative cisplatin dose of 200 mg/m^2^ body surface area. Thus, chemotherapy is not being modified within this de-escalation trial.

Serious adverse events are defined according to CTCAE criteria and reported without delay to the study coordination centre. Reporting is performed via eCRFs within the central RadPlanBio platform, which automatically generates notifications to the study coordination centre, including the principal investigator.

In addition to the clinical assessment including outcome and toxicities, patients‘ quality of life is being captured and analysed before start of radio(chemo)therapy, at the end of radio(chemo)therapy as well as at follow-up visits using the QLQ-C30 and the QLQ-HN35 questionnaires of the EORTC in german language.

Patients are actively followed according to the study schedule. Missed visits are documented, and efforts are made to minimise loss to follow-up.

### Endpoints

The primary endpoint of this study is the number of locoregional recurrences. Secondary endpoints include acute toxicities as assessed by CTCAE 4.0, late toxicity 24 months after end of radio(chemo)therapy (CTCAE 4.0), quality of life (assessed prior to, during and up to two years after completion of radio(chemo)therapy), locoregional recurrence rates as well as overall survival after 60 months after end of radio(chemo)therapy.

Outcome assessments are performed according to predefined time points as specified in the study schedule.

### Translational research

Data obtained within this trial should serve for further translational research. Provided patients’ approval, tumour specimen are being collected (as formalin-fixed paraffin-embedded material). If feasible at the respective study center, also blood samples prior to start of radio(chemo)therapy and at the end of the second week of the treatment are being collected, e.g. for evaluation of prognostic markers such as circulating tumour DNA and identification of novel markers or therapeutical targets.

### Statistics

We calculated stopping criteria, i.e. the number of recurrences, for which the trial has to be stopped, considering (interim) analyses after 10, 20 and 30 patients have completed 2-year follow-up. Assumptions are: expected recurrence rate: 0.03 in the de-escalation arms, maximum tolerated recurrence rate: 0.1, probability of stopping the study in error despite the expected recurrence rate is achieved: α < 0.1, probability of correctly stopping the study if the recurrence rate is too high: 1-β > 0.6. The calculated numbers that lead to the study being stopped are: 2 recurrences among the first 10 patients, 3 recurrences among the first 20 patients, 3 recurrences among the first 30 patients. The calculation was performed using the toxbdry function of the clinfun package in R statistics software, which applies a Pocock-type boundary for repeated testing [36–38].

For each of the two interventional arms, 60 patients per risk-profile are necessary (*n* = 120 patients in total), i.e. 30 patients in dose-de-escalation step 1 and 30 patients in dose-de-escalation step 2. After closure of the first dose de-escalation step and before opening of the second dose de-escalation step, patients are being enrolled in the respective observational arm according to their risk profile.

It has been assumed that about 50% of the OPSCC are HPV-driven (defined by p16 overexpression and HPV16 DNA positivity). In addition, it is supposed that 6% of the patients will die before the 24 months follow up has been reached or will withdraw from the study during follow-up. In addition, a dropout rate of 15% is assumed. This leads to 152 patients to be enrolled in the HPV-positive interventional arms. In parallel, about 152 patients will be enrolled in the respective observational arms including drop-outs. The total patient number is thus 304. Additional patients will be enrolled in the observational arm between closure of dose step 1 and opening of dose step 2.

The number of patients in the observational arms is not restricted. The observational arm closes when the interventional arms of the second dose de-escalation step closes.

Missing data will be handled using appropriate statistical methods depending on the type and extent of missingness. Sensitivity analyses may be performed if applicable.

Additional subgroup analyses may be performed for the high-risk and intermediate-risk cohorts.

### Data management and monitoring

All study data are entered into a centralised, secure electronic database (RadPlanBio platform). Data quality is ensured by predefined validation rules, monitoring, and regular data checks.

An independent data monitoring committee (DMC) has been established. The DMC is independent of the study investigators and oversees patient safety and trial conduct based on predefined stopping rules. The committee regularly reviews safety data and trial progress.

Important protocol modifications (e.g. changes to eligibility criteria, outcomes or analyses) will be communicated to the responsible ethics committees, participating study centres, and trial registries in accordance with regulatory requirements. The trial registry entry will be updated accordingly. Where applicable, trial participants will be informed.

Personal information of participants will be collected and processed at the participating centres in accordance with applicable data protection regulations. All data will be recorded in a pseudonymised form using unique study identifiers. Access to identifiable data will be restricted to authorised study personnel.

Data will be stored securely in the RadPlanBio platform and handled confidentially throughout the study and after its completion. Any data sharing will be conducted in a pseudonymised form to ensure participant confidentiality.

### Trial governance: roles and responsibilities

The DELPHI trial is led by the principal investigator (PI), M.K. The trial sponsor is the Dresden University of Technology, Faculty of Medicine and University Hospital Carl Gustav Carus, Fetscherstraße 74, 01307 Dresden, Germany. The study protocol was developed by the protocol committee consisting of M.B., M.K., F.L., E.G.C.T., A.L., S.A., and A.A. Administrative study coordination is performed by S.K. and D.B. Statistical design and biometrical oversight are provided by S.L. Participating centres are responsible for patient recruitment, treatment and data acquisition according to the study protocol. Central pathology, including HPV testing, is performed at the Institute of Pathology at the University Hospital Carl Gustav Carus, Dresden. Data management is coordinated centrally using the RadPlanBio platform.

### Planned timeline

The primary endpoint (locoregional control) is being achieved after 24 months after end of radio(chemo)therapy. The secondary endpoints are being assessed up to 60 months after end of therapy. This indicates that the follow-up for the primary endpoint is reached 24 months after the last patient enrolled has completed radio(chemo)therapy, whereas the follow-up for secondary endpoints is reached 60 months after the last patient enrolled has finished radio(chemo)therapy. Recruitment into this study started in December 2018. It is assumed that the last patient will be recruited in the second dose-de-escalation step by end of November 2027, i.e. the last study visit for assessment of the primary endpoint is in November 2029, and for the secondary endpoints in November 2032.

### Perspective

At the end of the second dose de-escalation step, or in case of reaching stopping criteria, the study group will decide for both risk groups whether step-wise treatment de-escalation is to be continued under use of further biomarkers (e.g. [39, 40]) combined with HPV-positivity or whether a randomised trial testing one of our reduced dose levels versus standard treatment is initiated.

## Supplementary Information

Supplementary Material 1.

Supplementary Material 2.

## Data Availability

The study protocol is described in this publication. Individual participant-level data and statistical code are not planned to be made publicly available due to data protection and privacy considerations. Pseudonymised data may be made available to qualified researchers upon reasonable request and are subject to applicable data protection regulations and approval by the study investigators.
