# Supplementary material for: De-escalation of adjuvant radio(chemo)therapy for patients with HPV-positive head and neck squamous cell carcinoma: study protocol for a phase I trial to reduce late toxicity (DELPHI)
Source: BMC Cancer. 2026 Apr 29;26:542. doi: 10.1186/s12885-026-16050-x (PMC13130450; doi:10.1186/s12885-026-16050-x)
Supplement: Supplementary file 1 — Supplementary Material 1. [file 12885_2026_16050_MOESM1_ESM.docx]

|  | **STUDY PERIOD** | | | | | | | | | | | | | | | | | | |
| --- | --- | --- | --- | --- | --- | --- | --- | --- | --- | --- | --- | --- | --- | --- | --- | --- | --- | --- | --- |
|  | **Screening** | **Baseline** | **Intervention period (RT week 1-6)** | | | | | | **RT end** | **Follow-up (post-treatment)** | | | | | | | | | |
| **TIMEPOINT** | **After surgery** | **0** | **1** | **2** | **3** | **4** | **5** | **6** | **7** | **6w** | **3m** | **6m** | **9m** | **12m** | **15m** | **18m** | **21m** | **24m** | **Every 6 m until 60 m** |
| **ENROLMENT:** |  | | | | | | | | | | | | | | | | | | |
| Eligibility assessment | X |  |  |  |  |  |  |  |  |  |  |  |  |  |  |  |  |  |  |
| Informed consent | X |  |  |  |  |  |  |  |  |  |  |  |  |  |  |  |  |  |  |
| Central HPV testing | X |  |  |  |  |  |  |  |  |  |  |  |  |  |  |  |  |  |  |
| Risk stratification and allocation to study arms |  | X |  |  |  |  |  |  |  |  |  |  |  |  |  |  |  |  |  |
| **INTERVENTIONS:** |  | | | | | | | | | | | | | | | | | | |
| Interventional arm A  (RT dose de-escalation, step 1) |  |  |  |  |  |  |  |  |  |  |  |  |  |  |  |  |  |  |  |
| Interventional arm B  (RT dose de-escalation, step 2) |  |  |  |  |  |  |  |  |  |  |  |  |  |  |  |  |  |  |  |
| Observational arm  (Standard RT) |  |  |  |  |  |  |  |  |  |  |  |  |  |  |  |  |  |  |  |
| Cisplatin* |  |  | X | X | X | X | X | (X) |  |  |  |  |  |  |  |  |  |  |  |
| **ASSESSMENTS:** |  | | | | | | | | | | | | | | | | | | |
| Toxicity |  | X | X | X | X | X | X | X | X | X | X | X | X | X | X | X | X | X | X |
| Concomitant medication |  | X |  |  |  |  |  |  | X | X | X | X | X | X | X | X | X | X | X |
| Quality of life  (EORTC QLQ-C30 and HN35) |  | X |  |  |  |  |  |  | X |  | X | X |  | X |  |  |  | X | X** |
| Examination by H&N specialist |  |  |  |  |  |  |  |  |  |  | X | X | X | X | X | X | X | X | X |
| Imaging |  |  |  |  |  |  |  |  |  |  | X |  | X |  | X |  | X |  | X*** |
| Locoregional control |  |  |  |  |  |  |  |  |  |  |  |  |  |  |  |  |  |  |  |
| Overall survival |  |  |  |  |  |  |  |  |  |  |  |  |  |  |  |  |  |  |  |

* Cisplatin-based radiochemotherapy only in high-risk arm (cumulative dose: 200 mg/m^2^ body surface area)

** every 12 months starting 36 months after end of radiotherapy

*** at least once a year or as determined by the treating physician

w = week, m = month, RT = radiotherapy
